# Optimal Placement of Virtual Masses for Structural Damage Identification

**DOI:** 10.3390/s19020340

**Published:** 2019-01-16

**Authors:** Jilin Hou, Zhenkun Li, Qingxia Zhang, Runfang Zhou, Łukasz Jankowski

**Affiliations:** 1 Department of Civil Engineering & State Key Laboratory of Coastal and Offshore Engineering, Dalian University of Technology, Dalian 116024, China; lizhenkunn@gmail.com (Z.L.); zhou0430@126.com (R.Z.); 2 Department of Civil Engineering, Dalian Minzu University, Dalian 116650, China; zhangqingxia_hit@hotmail.com; 3 College of Urban and Rural Construction, Shanxi Agricultural University, Jinzhong 030801, China; 4 Institute of Fundamental Technological Research, Polish Academy of Sciences, Warsaw 02-106, Poland; ljank@ippt.pan.pl

**Keywords:** damage identification, sensor optimization, Virtual Distortion Method (VDM), Particle Swarm Optimization (PSO) algorithm, sensitivity

## Abstract

Adding virtual masses to a structure is an efficient way to generate a large number of natural frequencies for damage identification. The influence of a virtual mass can be expressed by Virtual Distortion Method (VDM) using the response measured by a sensor at the involved point. The proper placement of the virtual masses can improve the accuracy of damage identification, therefore the problem of their optimal placement is studied in this paper. Firstly, the damage sensitivity matrix of the structure with added virtual masses is built. The Volumetric Maximum Criterion of the sensitivity matrix is established to ensure the mutual independence of measurement points for the optimization of mass placement. Secondly, a method of sensitivity analysis and error analysis is proposed to determine the values of the virtual masses, and then an improved version of the Particle Swarm Optimization (PSO) algorithm is proposed for placement optimization of the virtual masses. Finally, the optimized placement is used to identify the damage of structures. The effectiveness of the proposed method is verified by a numerical simulation of a simply supported beam structure and a truss structure.

## 1. Introduction

Nowadays, structural damage identification becomes a significant field in Structural Health Monitoring (SHM), and many new ideas are proposed in a growing number of studies. Spencer Jr. et al. [1] reviewed recent advances in wireless smart sensors for multi-scale monitoring and control of civil infrastructure. An et al. [2] proposed a novel method for computing the curvature directly from acceleration signals without identifying the modal shapes of the structure. Two examples were adopted to verify the effectiveness of the method, and its robustness to measurement noise. Hu et al. [3] reported on structural health monitoring of a prestressed concrete bridge based on statistical pattern recognition of continuous dynamic measurements over 14 years. Laflamme et al. [4] developed a soft capacitive sensor for structural health monitoring of large-scale systems; the performance of the sensor was then characterized for applications in dynamic vibration-based monitoring [5]. Yang et al. [6] proposed two methods for damage identification of a bridge based on measurements by a test vehicle. Fu [7] used wireless smart sensors to identify modes of structures to monitor sudden events in civil infrastructure. Li et al. [8] monitored fatigue cracks in steel bridges using a large-area strain sensing technology. Structural modes are the most basic characteristics of structures, and the approaches based on modal information are among the most commonly used methods of structural damage identification. Pnevmatikos et al. [9] introduced wavelet analysis for damage detection of a steel frame structure with bolted connections, and the presented experiment showed the effectiveness of the wavelet approach to damage detection of frame structures assembled using bolted connections. Ubertini [10,11] proposed an automated output-only modal identification procedure and utilized carbon nanotube cement-based sensors to identify natural frequencies of a reinforced concrete beam. Xu et al. [12] used embedded piezoceramic transducers to identify damage of a concrete column subject to blast loads. Zhang et al. [13] identified damage of concrete-filled square steel tube (CFSST) column joints under cyclic loading. Ginsberg et al. [14] identified damage parameters of framework by combining sparse solution techniques with an Extended Kalman Filter. The measurement equation was expanded by an additional nonlinear L1-minimizing observation to ensure sparsity of the damage parameter vector. Jiang et al. [15] monitored fatigue damage of modular bridge expansion joints using piezoceramic transducers. Zhang et al. [16] verified a method for concrete strength validation by smart aggregate-based stress monitoring. Most of these approaches are related to modes of structures. However, the modes that can be identified in real application usually do not convey enough information for full characterization of the monitored structure, and they are practically always insensitive to local damage. Researchers have thus proposed methods based on adding components such as mass and stiffness to the structure that can effectively increase the amount of modal information and improve the accuracy of damage identification. Nalitolela et al. [17] proposed a model updating method that adds various physical masses or stiffeners to the structure and utilizes modal information of the updated structures. Then, an improved method was proposed by adding imaginary masses to the preselected degrees of freedom (DOFs) [18]. In 2010, Dems and Mroz [19] further added controllable parameters such as supports, loads and temperature to the original structure, and identified the damage by modal, static and thermodynamic methods. Lu [20] took the beam structure as an example, and comprehensively analyzed the influence of the value, position and the number of the additional masses on damage identification in the additional mass method. Hou et al. [21] derived the virtual mass equation using structural excitation and response based on the VDM, and the effectiveness of the method was verified by an experiment of the frame structure. Therefore, adding virtual masses on structures is an efficient way to obtain more information related to natural frequencies for damage identification. However, there are few studies on the optimal placement of masses and other physical parameters. In fact, the value, positions and the number of the additional virtual masses can greatly affect damage identification results, so the optimal placement of additional virtual masses is the main prerequisite for the accuracy of structural damage identification. Therefore, the problem of optimal placement of virtual masses for the purpose of structural damage identification is studied in this paper.

The problem of optimal placement of virtual masses is similar to the problem of optimal sensor placement, so that similar methods might be applied for this research. In this paper, the optimization criterion and the algorithm for the optimal placement of virtual masses are along the lines provided by the research on the optimal placement of sensors.

There are three criteria that are mainly used: the minimum transmission error criterion [22], the modal kinetic energy criterion [23] and the model reduction criterion [24]. The basic theory behind the minimum transmission error approach is to use the unbiased estimation of the system parameter identification error. When the trace or the determinant of the Fisher information matrix reaches its maximum value, the system parameter identification error reaches its minimum accordingly. Kammer [22] applied the Fisher information matrix to the sensor placement problem for identification of structural modal parameters, and proposed the famous Effective Independence (EI) method, which eliminates in a stepwise manner the DOFs that contribute little to the linear independence of the target mode vectors by maximizing the determinant of the information matrix. Zhan [25] used the modal strain energy method to modify the EI method and applied it to the sensor optimal placement of the truss bridge structure. Yi [26] proposed a new multi-dimensional sensor optimization layout criterion combined with the EI method and the mode assurance criterion, and introduced the Wolf Group algorithm to improve the computational efficiency. Zhang [27] proposed an effective independence–total displacement method to address the problem of optimal sensor placement in hydraulic structures. These sensor optimization studies are in general based on modal observability. Silvers [28] proposed an optimization method, which optimized the sensor arrangement by maximizing the sensitivity of the natural frequency to the damage. Bruggi [29] proposed a method for sensor placement optimization to identify the damage of flexible plates. Li [30] used Non-dominated Sorting Genetic Algorithm II (NSGA-II) and wavelet decomposition to analyze and optimize sensor distribution for structural impact monitoring. The general purpose of the minimum transmission error criterion is to make the modal matrix include as much information as possible, while the purpose of adding masses is to improve the damage identification accuracy. Therefore, this paper draws on the construction of the Fisher information matrix in the transmission error criterion, and it proposes an optimization criterion based on the sensitivity information matrix in damage identification. The aim is to obtain a sensitivity matrix that contains as much information as possible.

After establishing the sensor optimization criterion, the next step is to select the optimization method to find the optimal solution under the corresponding criterion. The current optimization algorithms can be classified as classical optimization algorithms and meta-heuristic algorithms. Classical optimization algorithms utilize classical approaches like the Newton method or the conjugate gradient method to optimize the placement of the measurement points. The optimization efficiency of these methods is relatively high, but they perform an intrinsically local search, so that the globally optimal solution might be difficult to find. The most known meta-heuristic algorithms include genetic, simulated annealing, particle swarm and cross entropy optimization algorithms. They are designed to be global and can thus effectively avoid falling into a locally optimal solution. The Particle Swarm Optimization (PSO) algorithm belongs to the global, meta-heuristic approaches. It was proposed by Kennedy and Eberhart [31] in 1995. The method utilizes a large number of search points treated as particles flying through the search space (particle swarm), which attracted to the optimal solution by changing their velocity based on the individual, local and global experiences. The PSO algorithm has the advantages of a fast convergence, few tunable parameters and an easy implementation. It is widely used in optimization calculations in various fields such as power design, intelligent control, and transportation. He et al. [32] used an improved PSO algorithm to solve the problem of multi-dimensional sensor layout based on information redundancy. The efficiency of the method was verified by taking the Laxiwa arch dam of the upper Yellow River as an example. Zhang [33] proposed an approach for optimal sensor placement based on the PSO algorithm for the structural health monitoring of long-span cable-stayed bridges, and established the fitness function to solve the optimal problem by using the root mean square (RMS) value of the non-diagonal elements in the modal assurance criterion matrix. For applications to discrete optimization variables, Kennedy et al. [34] proposed a binary PSO algorithm for 0–1 programming problems. The particle position was represented by a binary variable, and the velocity of the particle meant the probability of taking 1 as the binary variable.

This paper takes the identification of damage parameters as the ultimate goal and studies the problem of optimal placement of the added virtual masses. It is structured as follows: Firstly, an optimization criterion based on the volumetric maximum of the sensitivity matrix is proposed. Secondly, due to the advantages of a low number of parameters and a small computational cost [35], the PSO algorithm is improved and applied for the optimal placement of virtual masses. Then, the value and the number of virtual masses is optimized. Thirdly, according to the optimization result, the virtual masses are arranged on the considered structure and damage identification is conducted by employing the sensitivity method. Finally, the feasibility and effectiveness of the proposed method are validated by a numerical simulation example of a simply supported beam structure and a truss structure.

## 2. The Effect of an Added Virtual Mass

In this method, the structure without additional masses is called the original structure, and the structure with an additional virtual mass is called the virtual structure. The notion “virtual” is used to emphasize that the influence of the additional mass is computed based on the recorded responses of the original structure, without mounting a real mass to the system.

Let the excitation be applied and the acceleration be measured in the same structural degree of freedom (DOF), and denote by h(ω) the corresponding (measured) acceleration frequency response of the original structure. Let a (virtual) mass be added in the same DOF and denote by H(ω,m) the corresponding acceleration frequency response of the virtual structure. The virtual mass is added just in one DOF, and the other DOFs remain unmodified, therefore the inertia force is generated just in the single involved DOF and it equals −mH(ω,m).

According to the basic theory of VDM [21], the influence of the additional mass can be equivalently modeled by its inertia force. Therefore, H(ω,m) can be expressed as the following sum of the original frequency response and the effects of the inertia force:

(1) H(ω,m)=h(ω)−mH(ω,m)h(ω)

This formula can be rearranged as:

(2) $$H\left(\omega ,m\right)=\frac{h\left(\omega \right)}{1+mh\left(\omega \right)}$$

In actual engineering projects, the frequency response is usually calculated by the Fourier Transform of time-domain excitations and responses. If the time-domain excitation is denoted by f(t), and the corresponding acceleration response in the same DOF is denoted by y(t), let A(ω) and F(ω) denote the corresponding frequency-domain signals obtained by the Fourier Transform. Then, by substituting h(ω)=A(ω)/F(ω) into Equation (2), one obtains the following simple formula for the acceleration frequency response of the virtual structure:

(3) $$H\left(\omega ,m\right)=\frac{A\left(\omega \right)}{F\left(\omega \right)+mA\left(\omega \right)}$$

Equation (2) can be used to determine the natural frequencies of the virtual structure, which can be then utilized for damage identification. It should be emphasized that the position and the direction of the applied excitation F(ω) and the measured acceleration response A(ω) should be the same, and that the virtual mass also must be added in the same position and direction. In other words, the virtual mass is constructed and added in the position where the sensor is.

## 3. Optimal Sensor Placement for Virtual Masses

In this section, the sensitivity information matrix is constructed by using the natural frequencies of the virtual structure with respect to the damage factor. Then the virtual mass optimization criteria are established based on the sensitivity information matrix. Finally, an optimization method for the virtual mass placement is proposed.

### 3.1. Sensitivity Information Matrix

It is assumed that there are n substructures in the structure to be identified, and that the damage factor $\mu_{l}$ of the *l*-th substructure represents its stiffness reduction ratio: it is equal to the stiffness ratio of the *l*-th substructure after damage to that of *l*-th substructure before damage. The global structural stiffness matrix after damage is expressed as K(μ), where:

(4) $$\mu =\left\{\mu_{1},\mu_{2},\ldots ,\mu_{n}\right\}$$

**Figure 1:**
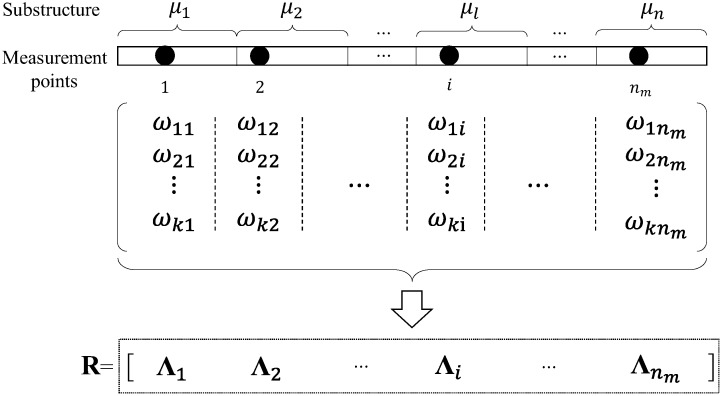
Construction of sensitivity information matrix.

As shown in Figure 1, it is assumed that there are *n_m_* available locations for virtual masses in the structure. And it is supposed that when the mass *m* is placed at the position *i* (*i* = 1, 2, …,*n_m_*), then the first *k* natural frequencies of *i*-th virtual structure can be identified as $\omega_{1i}\left(\mu ,m\right),\;\omega_{2i}\left(\mu ,m\right),\;\ldots ,\;\omega_{ki}\left(\mu ,m\right)$. The *j*-th natural frequency and the mass normalized mode of the *i*-th virtual structure are denoted thus by $\omega_{ji}\left(\mu ,m\right)$ and $\Psi_{ji}\left(\mu ,m\right),$ respectively.

Because the larger order of the natural frequency is, the larger its absolute identification error will be, the relative sensitivity is adopted for analysis. The relative sensitivity $r_{ji,l}$ is the normalized gradient of $\omega_{ji}\left(\mu ,m\right)$ with respect to the damage factor $\mu_{l}$:

(5) $$r_{ji,l}\left(\mu ,m\right)=\frac{1}{\omega_{ji}\left(\mu ,m\right)}\frac{\partial \omega_{ji}\left(\mu ,m\right)}{\partial \mu_{l}}=\frac{\Psi_{ji}^{T}\left(\mu ,m\right)K_{l}\Psi_{ji}\left(\mu ,m\right)}{2\omega_{ji}^{2}\left(\mu ,m\right)}$$

When the mass *m* is added in the *i*-th measuring point, the relative sensitivity of the *j*-th natural frequency to all *n* substructure damage factors μ can be arranged as the following vector:

(6) $$r_{ji}\left(\mu ,m\right)=\frac{1}{\omega_{ji}\left(\mu ,m\right)}\frac{\partial \omega_{ji}\left(\mu ,m\right)}{\partial \mu }={\left\{r_{ji,1},r_{ji,2},\ldots ,r_{ji,n}\right\}}^{T}$$

Furthermore, for the *i*-th measurement point, the relative sensitivity information of all *k* natural frequencies with respect to all *n* substructure damage factors can be arranged as a single vector $\Lambda_{i}={\left\{r_{1i}^{T},r_{2i}^{T},\ldots ,r_{ki}^{T}\right\}}^{T}$, which is a column vector with *kn* elements. The sensitivity information matrix R of the structure is arranged as shown in Equation (7), and it contains *kn* rows and $n_{m}$ columns:

(7) $$R=\left\{\Lambda_{1},\Lambda_{2},\ldots ,\Lambda_{n_{m}}\right\}$$

In the conventional sensitivity matrix, generally, each column vector represents the sensitivities of all modal information with respect to one considered parameter. In this paper, each column vector of the sensitivity matrix R represents the sensitivities of all modal information with respect to all considered parameters obtained by adding a virtual mass in one point. This new arrangement is more conducive to the analysis of the correlation between points.

### 3.2. Optimization Criterion

The optimal placement of virtual masses is to ensure the accuracy of damage identification, so the optimization criterion should assess two conditions: first, the sensitivity for each measurement point should be relatively high; second, sensitivity information for different measurement points should be as irrelevant as possible. The Volumetric Maximum Criterion can guarantee both of the above conditions. The geometric meaning of the optimization criterion based on the volumetric maximum criterion of the sensitivity matrix is described below.

**Figure 2:**
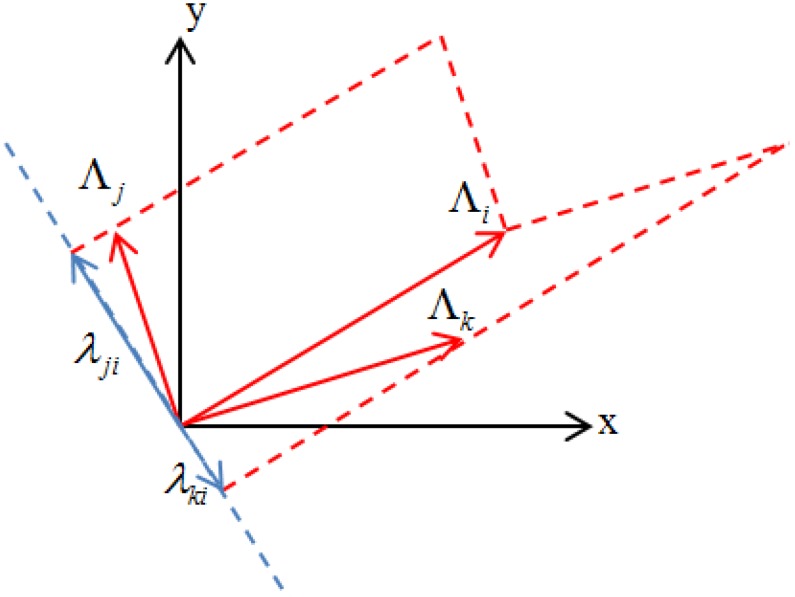
Schematic diagram of the optimization criterion.

As shown in Figure 2, the vectors $\Lambda_{i}$, $\Lambda_{j}$ and $\Lambda_{k}$ represent the sensitivity information vectors of the *i*-th, *j*-th and *k*-th measurement points in the sensitivity matrix R, respectively. In this figure, the modulus of the *i*-th measurement point sensitivity information vector $\Lambda_{i}$ is maximum. $\Lambda_{i}$ can be regarded as the vector of first selected point. To determine the next point, the vector that is the most irrelevant to the *i*-th vector is selected from among the *j*-th and *k*-th vectors. Figure 2 shows that the irrelevance between the vectors $\Lambda_{i}$ and $\Lambda_{j}$ is obviously greater than that between the vectors $\Lambda_{i}$ and $\Lambda_{k}$.

Moreover, the component $\lambda_{ji}$ of the vector $\Lambda_{j}$ in the subspace perpendicular to $\Lambda_{i}$ is larger than $\lambda_{ki}$, which is the component of $\Lambda_{k}$ in the subspace perpendicular to $\Lambda_{j}$. Obviously, the area formed by the vectors $\Lambda_{i}$ and $\Lambda_{j}$ is larger than that formed by $\Lambda_{i}$ and $\Lambda_{k}$. Therefore, the area can be used to describe the irrelevance between two vectors. If this situation is extended to a 3-dimensional or a higher dimensional space, it can be concluded that the greater the irrelevance of the vectors, the larger the volume. Therefore, the volume of the formed parallelogram can be used as the criterion for evaluating the irrelevance of the vectors in the matrix. Consequently, maximization of the volume of the sensitivity matrix can be used as the criterion for the optimal placement of the virtual masses.

To meet the two above conditions, the corresponding objective function based on the volumetric maximum criterion of the sensitivity matrix can be expressed as:

(8) $$f_{1}\left(\pi ,m\right)=V\left(R\left(\pi ,m\right)\right)$$

where π represents the location layout scheme of the virtual masses, R(π,m) represents the structural sensitivity matrix under the corresponding placement scheme of the virtual masses, and *V* represents the volume formed by the column vectors of measurement points in the sensitivity matrix. When the sensitivity matrix R contains only one vector, *V* is the length of that vector; when R contains 2 vectors, *V* is the area formed by two vectors; and when R contains three or more vectors, *V* can be understood as the volume or the generalized volume formed by the vectors, and its volume can be obtained by Equation (9), where $\det\left(R^{T}R\right)$ represents the determinant of matrix $R^{T}R$:

(9) $$V\left(R\right)=\sqrt{\det\left(R^{T}R\right)}$$

In the application of this method, if $\det\left(R^{T}R\right)>0$, then the number $n_{m}$ of the measurement points should meet the condition $n_{m}\leq kn$, where *k* is number of the identified modes and *n* is the number of the substructures. Given $F=R^{T}R$, then F is defined as the Fisher information matrix in this method. Therefore, finding the volume of the sensitivity matrix R is the problem of finding the determinant of the Fisher information matrix F.

In the process of optimization, it is actually the process of finding the extreme minimum value of the objective function, so Equation (8) can be revised to Equation (10):

(10) $$f_{2}\left(\pi ,m\right)=-\sqrt{\det\left(F\right)}$$

### 3.3. Placement Optimization of Virtual Masses

The variables to be optimized in this paper include the value, the number, and the positions of the virtual masses. It is difficult to simultaneously optimize three variables of different characters, so they are optimized separately. Firstly, the sensitivity analysis and error analysis are used to optimize the value of the virtual mass, and then the positions are optimized. Finally, the number of virtual masses is discussed.

#### 3.3.1. Preliminary Optimization of the Value of the Additional Virtual Mass

The purpose of adding virtual masses is to improve the sensitivity of the modal information to local damage. Therefore, the sensitivity analysis of the finite element model is used to determine the value of the additional masses, and Equation (3) shows that the additional virtual masses may cause errors of the frequency response. It may in turn result in errors of the identified natural frequency, thereby reducing the accuracy of the damage identification. Therefore, two factors in the selection of the virtual masses should be considered: the sensitivity and the frequency identification error.

The influence of the additional virtual mass value on the frequency identification error is studied by adding mass to the SDOF (single-degree of freedom) structure. The physical parameters of the structure are assumed as follows: M=1, K=1, C=0. Then the natural frequency of the original structure $\omega_{0}=1$. Measurement error of frequency response are denoted by Δ. When a unit impulse excitation is applied to the original structure, the acceleration frequency response can be expressed as Equation (11):

(11) $$h\left(\omega \right)=\frac{\omega^{2}}{\omega^{2}-1}+\Delta$$

Substituting h(ω) into Equation (2), the acceleration frequency response H(ω,m) of the virtual structure with the additional virtual mass *m* can be easily obtained, and then the corresponding natural frequency $\omega_{e}$ can be estimated by peak-picking, which is shown in Equation (12):

(12) $$\omega_{e}=\sqrt{\frac{1+m\Delta }{1+m+m\Delta }}$$

After adding mass *m*, the accurate natural frequency $\omega_{a}$ is preliminarily estimated using the stiffness and mass of the structure, which is shown in Equation (13):

(13) $$\omega_{a}=\sqrt{\frac{1}{1+m}}$$

The relative error of the estimated natural frequency with respect to the accurate natural frequency can be expressed by substituting Equations (12) and (13) into Equation (14):

(14) $$\delta =\frac{\omega_{e}-\omega_{a}}{\omega_{a}}$$

**Figure 3:**
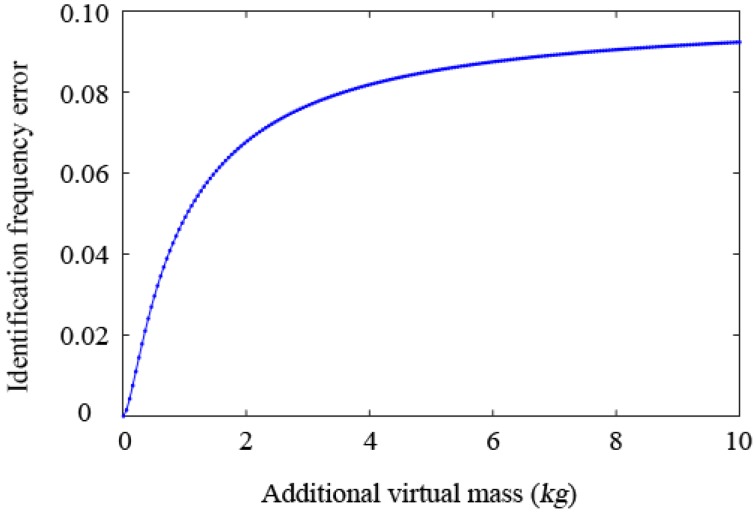
Relationship between the relative frequency identification error and the additional mass.

By considering an example value Δ=0.1, the curve of the estimated relative error of the natural frequency of the SDOF system with the additional virtual mass *m* is drawn in Figure 3. It can be seen that the frequency identification error increases with the increase of mass.

In conclusion, from the view of sensitivity analysis, additional masses can improve the sensitivity of the frequency information to local damage. From the view of errors, the greater the added mass is, the greater the error of the estimated natural frequency is. Therefore, the choice of the virtual mass should balance between these two factors, i.e., frequency sensitivity and estimated frequency error.

#### 3.3.2. Optimization Method for Virtual Masses Placement

There are many algorithms that can solve the considered optimization problem. Often, the PSO requires fewer parameters to be tuned and it takes less computational effort in comparison to other meta-heuristic algorithms. The PSO also has the advantages of a fast convergence and an easy implementation. The discrete PSO algorithm can find the global optimal solution in a straightforward procedure, and the calculation results are stable. In this paper, the PSO algorithm is modified and applied for the optimization of virtual masses.

In the PSO algorithm, a search point is treated as a particle that travels through the search space. Each such particle has its own position and velocity, which are modified in the successive optimization steps according to the corresponding fitness value. The PSO algorithm takes fitness function as the criterion to evaluate the quality of the solution in the process of searching for the optimum. Therefore, the selection of the fitness function directly affects the determination of the optimal solution. The optimization criterion based on the volumetric maximum of the sensitivity matrix ensures the maximum irrelevance between the sensitivity information of each measurement point by maximizing the volume of the sensitivity matrix, so that it contains as much information as possible.

The PSO algorithm with a linearly decreasing inertial weight is applied in this paper, and the iterative velocity update equation is as follows:

(15) $$v_{id}^{t+1}=wv_{id}^{t}+c_{1}r_{1}\left(p_{id}^{t}-x_{id}^{t}\right)+c_{2}r_{2}\left(p_{gd}-x_{id}^{t}\right)$$

where w is the inertial weight, $v_{id}^{t}$ is the velocity in the *d*-th dimension of the *i*-th particle during the *t*-th iteration; $c_{1}$ and $c_{2}$ are the acceleration coefficients (usually positive constants); $r_{1}$ and $r_{2}$ are random numbers uniformly distributed on [0,1]; $p_{id}^{t}$ is the position in the *d*-th dimension of the past individual best point of the *i*-th particle during the *t*-th iteration; $p_{gd}$ is the position in the *d*-th dimension of the best global extremum point (of the entire particle swarm).

The standard PSO algorithm is mainly applied to the optimization problem of continuous space functions. In this paper, the optimization problem of virtual mass placement is how to choose $n_{m}$ positions from *N* possible positions, which is a discrete problem. Therefore, this paper uses the discrete PSO algorithm to optimize the virtual mass placement [31]. The velocity update equation of the discrete binary algorithm is the same as in the original PSO algorithm, but the position update in this method is different and should be studied.

The location of the virtual masses is encoded in the binary code, and $x_{i}^{t}$ indicates the position of the *i*-th particle in the *t*-th generation. Each $x_{i}^{t}$ represents a solution to the optimization problem, $x_{i}^{t}=\left\{x_{i1}^{t},x_{i2}^{t},\ldots ,x_{iN}^{t}\right\}$, where *N* is the number of possible positions. If $x_{ij}^{t}=0$, the *i*-th particle does not arrange the virtual mass at the *j*-th position in the *t*-th iteration. Otherwise, when $x_{ij}^{t}=1$, it indicates that the *j*-th position is used to place the virtual mass. The velocity $v_{ij}^{t}$ represents the probability that the *j*-th binary bit is 1, therefore it is mapped to the interval [0,1]. The mapping method generally uses the sigmoid function as shown in Equation (16):

(16) $$s\left(v_{id}\right)=\frac{1}{1+exp\left(-v_{id}\right)}$$

where $s\left(v_{id}\right)$ is the probability that the position $x_{id}$ equals 1. In the traditional PSO algorithm, the selection of $x_{id}$ is based on Equation (17):

(17) $$x_{id}=\{\begin{array}{cc} 1 & \mathrm{if}\;\;\mathrm{rand}\left(\;\right)\leq s\left(v_{id}\right)\\ 0 & \mathrm{otherwise} \end{array}$$

where rand( ) is a random number uniformly distributed in [0,1]. However, the number of selected positions obtained this way (that is, the number of 1 s) is possibly not equal to the required number $n_{m}$. In this paper, the above methods is modified by ranking the difference value between $s\left(v_{id}\right)$ and the vector of all *N* random numbers rand( ) from large to small, and assigning 1 to the largest $n_{m}$ of them, so that always exactly $n_{m}$ measurement points are selected to place the virtual masses. As shown in Equation (18):

(18) $$x_{id}=\{\begin{array}{cc} 1 & \mathrm{if}\;\;\mathbb{R}\left(s\left(v_{id}\right)-\mathrm{rand}\left(\;\right)\right)\leq n_{m}\\ 0 & \mathrm{otherwise} \end{array}$$

where ℝ(·) represents the position number of the argument in the list of all arguments sorted in the descending order. For example, let $z_{i}=s\left(v_{id}\right)-\mathrm{rand}\left(\;\right)$, the variables $z_{1},z_{2},\ldots ,z_{N}$ are sorted in the descending order, and $\mathbb{R}\left(z_{i}\right)$ is the position of $z_{i}$ in the sorted list.

The main steps of discrete PSO algorithm is as follows:

(1) Set algorithm parameters;
(2) Initialize the position and the velocity of all particles. The position of each particle $x_{ij}^{0}$ is randomly generated to be 0 or 1, where $i=1,2,\ldots ,\bar{N}$ and i=1,2,…,N and $\bar{N}$ is the number of particles in the swarm. The velocity of the particle is generated as a random number between 0 and 1;
(3) Calculate the fitness value of each particle in the population, and compare the particle fitness value with its individual best value $P_{i}$. If it is better than $P_{i}$, then store the current position as $P_{i}$;
(4) Compare the best individual extremum value $P_{i}$ with the global extremum value $P_{g}$. If it is better than $P_{g}$, it is stored as an updated value of $P_{g}$;
(5) Update the velocity and the positions of the particle. The velocity of the particle can be updated according to Equation (15), and $s\left(v_{id}\right)$ can be calculated from Equation (16). The positions with the first $n_{m}$ maximum differences between $s\left(v_{id}\right)$ and rand( ) are be selected to place the virtual mass (Equation (18)), that is the corresponding bits are set to 1, while the others remain 0;
(6) Stop the operation when the number of iterations reaches a pre-set maximum number of iterations, and output $P_{g}$ and the corresponding fitness value, otherwise go to step 3.

#### 3.3.3. Determination of the Number of the Virtual Masses

The number of the virtual masses affects the accuracy of damage identification, so it is determined by analyzing and comparing the accuracy of the identified structural damage. The specific method is introduced in the numerical simulation.

## 4. Numerical Simulation

### 4.1. A Simply Supported Beam

**Figure 4:**
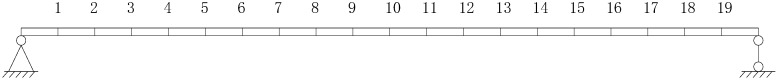
Finite element model of simply supported beam.

A simply supported beam is used to verify the effectiveness of the proposed virtual mass optimization method, see Figure 4. The span of the simply supported beam is 1 m, the width of the section is 0.05 m, the thickness of the section is 0.005 m, the elastic modulus of steel used in the structure is $2.1\times 10^{11}$ Pa, and the density of steel is used as $7.85\times 10^{3}$ kg/m^3^. The structure is divided into 20 finite elements. As shown in Figure 4, there are 19 vertical DOFs for candidate position of virtual mass, which are numbered as 1–9.

The structure is divided into 10 substructures, each substructure contains two finite elements. Structural damage is considered as follows: Substructure 3 and substructure 8 are damaged simultaneously, and their stiffness decreases by 20% and 30%, respectively. This damage scenario can be expressed as $\mu_{3}=0.8,\;\mu_{8}=0.7$.

Firstly, sensitivity analysis and frequency error analysis are used to preliminarily determine the value of the virtual masses. Then, an example of eight virtual masses placement is analyzed, and the improved discrete PSO algorithm is used to search for the optimal positions of virtual masses. Finally, the influence of the number of the masses on the variance of the identified damage factors is studied under 10 groups of noise, and the number of virtual masses is determined.

#### 4.1.1. Determination of Virtual Masses Value

The influence of sensitivity and frequency identification error should be considered in determining the value of virtual masses.

**Figure 5:**
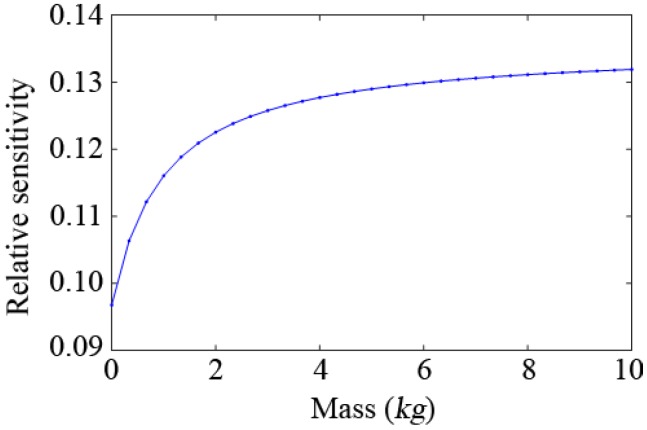
Relationship between the relative sensitivity of the first natural frequency and the additional mass.

Firstly, relative sensitivity analysis of the simple supported beam model shown in Figure 4 is performed. Different virtual masses are added to an arbitrary node of the structure (the 6th position is taken here as an example). By using Equation (5), the relative sensitivity of the first natural frequency of the structure with different virtual masses is obtained, and the result is shown in Figure 5. The relative sensitivity of the first natural frequency increases with the increase of the additional virtual mass.

**Figure 6:**
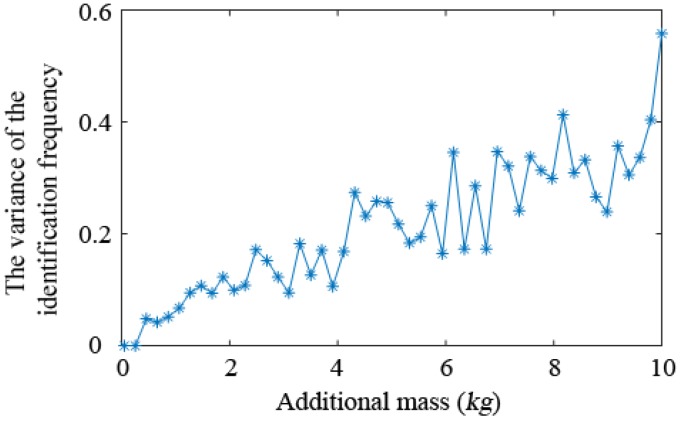
Variance of the identified natural frequency under different virtual mass.

The simply supported beam structure used in this paper is a multi-degree freedom (MDOF) system, and the relationship between the frequency identification error of the SDOF system and the additional virtual masses shown in Figure 3 is no longer applicable to the model. For the simply supported beam shown in Figure 4, the virtual masses are added to the 6th node of the structure, and the variance of the identified natural frequency is calculated after applying 20 groups of noise (5% white noise) on the test frequency response. After the structure is attached with different virtual masses, the variance of the identified natural frequency is shown in Figure 6. It shows that the error of the identified natural frequency increases as the virtual mass increases. When the mass is about 3 kg, the variance of the identified natural frequency is still relatively small, and the relative sensitivity is already high, so the virtual mass value is selected to be 3 kg.

#### 4.1.2. Virtual Masses Placement Optimization Using PSO Algorithm

The following presents the arrangement optimization of the virtual mass locations for 3 kg virtual mass. Firstly, the sensitivity matrix of the structure is calculated by Equation (5). Then the sensitivity values of the first 3 natural frequencies with respect to the damage factors of the 10 substructures are calculated when successively placing 3 kg mass in the 19 vertical DOFs. Finally, the sensitivity matrix is obtained according to Figure 1.

**Figure 7:**
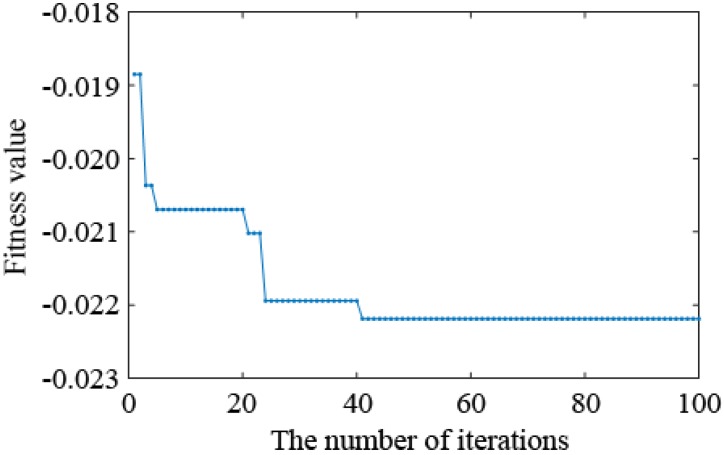
Variation curve of discrete PSO fitness function and iteration times.

**Figure 8:**
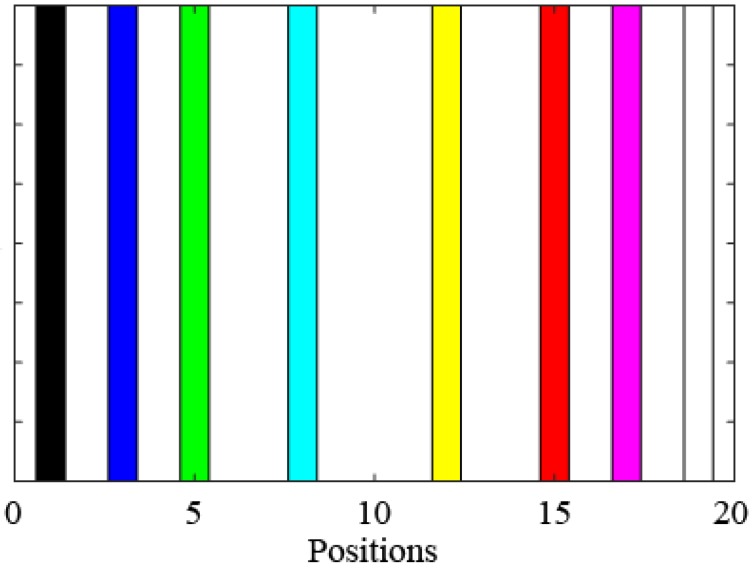
The optimization order of the measuring points.

The basic parameters of the PSO algorithm are shown in Table 1. Due to the randomness of the method, the optimization results might be different each time. In this paper, four random trials have been carried out and the results are shown in Table 2:

It can be seen from Table 2 that the optimized placements of the virtual masses and the best fitness values (Equation (10)) obtained in the 4 tests are exactly the same, but the optimization time differs slightly. To represent the iterative process of a particle, the relationship between the number of iterations and the fitness value for a test particle is drawn in Figure 7. The position distribution diagram of the eight measuring points in the optimized placement is shown in Figure 8. In Figure 7, the fitness function value decreases as the number of iterations increases. Past the 40th iteration, the fitness function value remains stable and no longer changes, which indicates that the improved PSO algorithm has converged. As seen in Table 2, the optimal values and the fitness values obtained in four random trials are identical, which indicates that the algorithm has found the global optimal solution, and thus proves the feasibility and efficiency of the improved PSO algorithm again. It can be seen from Figure 8 that the optimized positions of the measurement points are quite evenly dispersed, and the left and right symmetrical distribution form is centered on the mid-span, indicating that the results obtained by the PSO algorithm based on the maximum of the sensitivity matrix volume are reasonable.

The proposed algorithm required only 1 s to complete the search on a typical desktop hardware configuration. The discrete PSO algorithm can find the global optimal solution easily, and the calculation results are stable.

#### 4.1.3. Damage Identification

**Figure 9:**
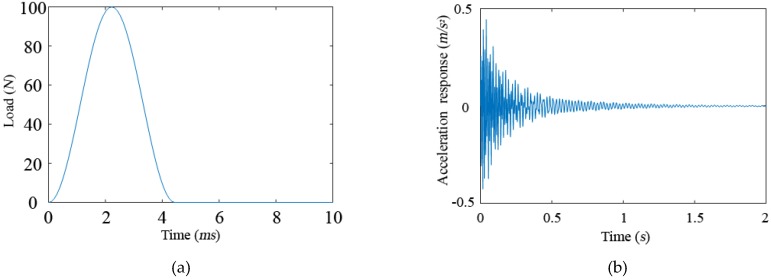
(**a**) Pulse excitation. (**b**) Acceleration response.

The excitation shown in Figure 9a is used to apply an impulse load. The load duration is 5 ms and the sampling frequency is 5000 Hz.

The influence of 5% white noise is considered. The excitation is applied to the 6-th position, and the corresponding acceleration response is shown in Figure 9b.

(1) Identification of the Natural Frequency with Added Virtual Masses

If the number of the added virtual masses is six, the positions can be optimized by the improved PSO algorithm, which is {1, 3, 8, 12, 17, 19}. The impulse excitation as shown in Figure 9a is applied to these six DOFs, and the acceleration responses in the same DOFs are calculated. The excitation and the response containing 5% noise are substituted into Equation (3), and the amplitude of the frequency response of each DOF is calculated after adding 3 kg of virtual mass. By using the Fourier transform and extracting the peak value, the first three natural frequencies of each DOF with added mass are obtained, as shown in Table 3. The finite element frequencies of the theoretical undamaged model after adding mass are shown in Table 4.

(2) Damage Identification

**Figure 10:**
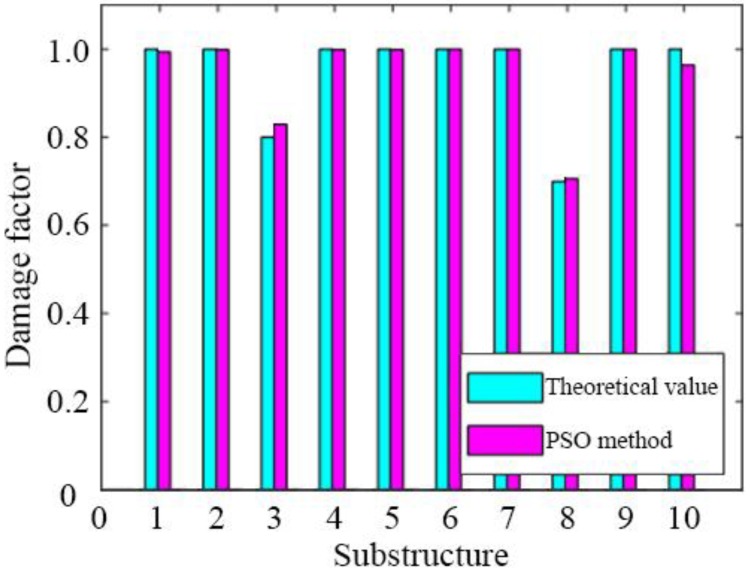
Identified damage factors.

The objective function for damage identification is easily built using the natural frequencies identified with added virtual masses and the frequency of the corresponding theoretical finite element model. The result of damage identification is shown in Figure 10, in which the abscissa is the substructure number and the ordinate is the damage factor.

The optimized virtual mass placement obtained by the discrete PSO algorithm can accurately identify the damage location and damage extent. Even in the case of 5% measurement noise, the identification results maintain good accuracy. The optimized arrangement scheme obtained by the discrete PSO algorithm based on the maximum volume of the sensitivity matrix is used for damage identification. Damage identification errors of substructure 3, substructure 8 and substructure 10 are 2.68%, 0.5% and 4.08%, respectively. This method has a high computational efficiency and can find the global optimal solution with a great probability. Moreover, the results fully meet engineering accuracy requirements.

#### 4.1.4. Determination of Virtual Masses Quantity

**Figure 11:**
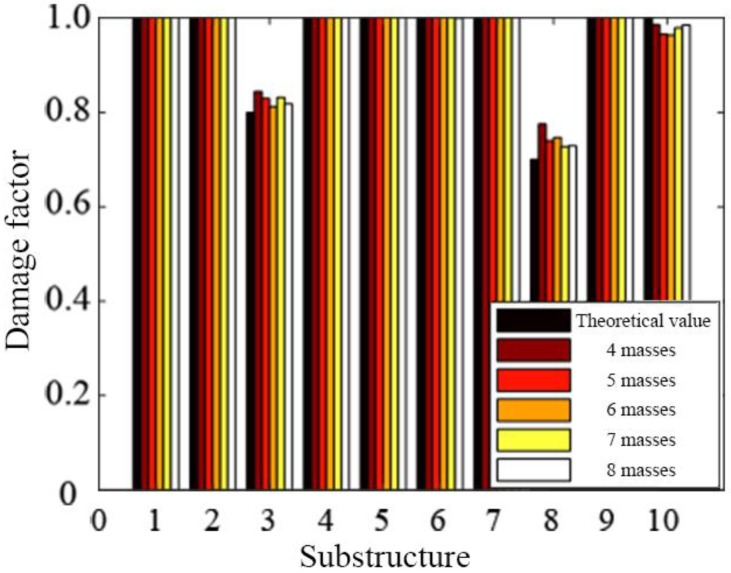
Damage identification results when arranging different numbers of masses.

When different number of virtual masses is placed on the beam, the identified results are different, as shown in Figure 11. It can be seen from Figure 11 that the larger the number of the virtual masses is, the closer the identified damage factor approaches the theoretical value. The identification error is the largest when four masses are arranged, and it decreases gradually with the increase of the number of virtual masses.

**Figure 12:**
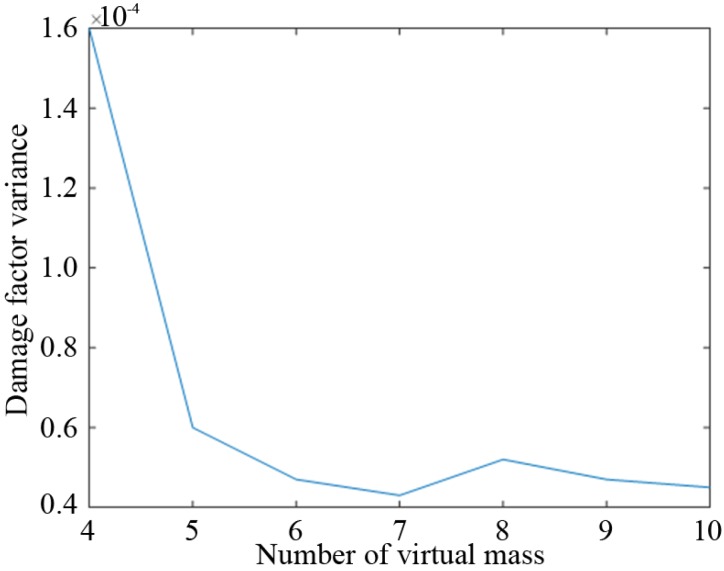
Variance of the damage factor when using different numbers of masses.

Then, in the case of applying 10 groups of noise (5% white noise), when 4–10 virtual masses are optimally placed, the variance results of the damage factors are shown in Figure 12.

The variance of the damage factors decreases with the increasing number of the virtual masses. When there are more than six masses, the variance of the damage factors tends to be stable and fluctuates only slightly. Overall, the number of the virtual masses is selected as six, the mass value is 3 kg, and the optimized placement is {1, 3, 8, 12, 17, 19}.

### 4.2. A Truss Structure

**Figure 13:**
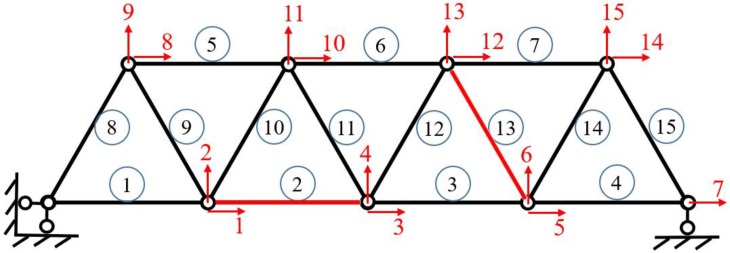
Truss structure model.

Figure 13 shows a truss structure model consisting of 15 members and nine nodes. The length of every member is 3 m, the height of the truss is 2.6 m, the elastic modulus of the rod is $2.0\times 10^{11}$ Pa, the density is $7.8\times 10^{3}$ kg/m^3^; the members are round steel tubes, the diameter of the steel tubes is 0.1 m, and the wall thickness is 0.05 m. Each member of the truss structure is a single element, so there are 15 elements and 15 DOFs.

Figure 13 shows the numbers of elements and DOFs. It is assumed that the members numbered 2 and 13 are damaged, and the damage factors are 0.6 and 0.7, respectively. The first four natural frequencies of structure with added masses are used as the basis for damage identification. The natural frequencies of the original structure and the damaged structure are shown in Table 5:

Firstly, the additional virtual mass of this truss structure is determined to be 200 kg based on the structural sensitivity analysis. The truss structure has a total of 15 DOFs, and they are all used as candidate positions for additional masses. Seven cases are considered by adding 4–10 virtual masses in the structure. The improved PSO algorithm is used to determine the optimum placements of the virtual masses, which are shown in Table 6.

The basic parameters in the PSO are the same as in Section 4.1.2. From the optimized results, all cases include lateral DOFs and longitudinal DOFs, and the positional arrangement is relatively scattered, which seems reasonable as assessed using the engineering common sense.

**Figure 14:**
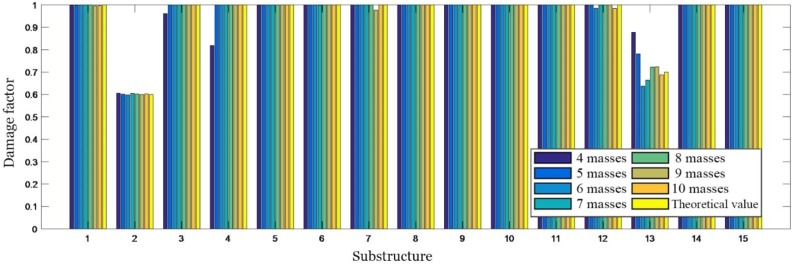
Damage identification results obtained by employing different numbers of masses.

Numerical simulations are performed for each of the seven cases listed in Table 6, and 5% white noise is considered in dynamic simulation. The natural frequencies of the damaged structure with added virtual masses are used for damage identification, and the identified damage factors are shown in Figure 14.

It can be seen from Figure 14, that when only four masses are added to the structure, there are some relatively large errors in the results of damage identification. This is because once a mass is added, four natural frequencies can be obtained. Therefore, four masses correspond to 16 natural frequencies used for damage identification. However, the structure has a total of 15 damage factors to be identified, and 15 parameters cannot be identified accurately by using only 16 natural frequencies due to the influence of measurement noise. When five masses are added to the structure, the damage can be relatively accurately localized. As the number of the added masses increases, more frequency information can be obtained, and the accuracy of damage identification is relatively high. When the number of the added masses is greater than seven, the identified damage factors have a slight fluctuation, but tend to be stable. The identified damage factors are very close to the actual values, which is enough to meet the engineering accuracy.

## 5. Conclusions

This paper proposes an optimal placement method of virtual masses for the purpose of damage identification. Firstly, a specific form of the sensitivity matrix is established, which is more conducive to the analysis of the correlation between points. Then, an optimization criterion called Volumetric Maximum Criterion is proposed, which is based on the volumetric maximum of the sensitivity matrix. This criterion promotes sensitivity matrices (and the corresponding placements) that contain more information that can be used to identify structural damage. Thereupon, an improved version of the PSO algorithm is proposed for optimization. Finally, the optimal placement of virtual masses is verified in numerical examples of a simply supported beam and a truss structure. It can be concluded that:

1. The Volumetric Maximum Criterion uses volume to quantify the information contained in the sensitivity matrix. It can ensure that the sensitivity matrix contains as much sensitivity information as possible, thereby ensuring the accuracy of damage identification;
2. The improved PSO algorithm is accurate and efficient for optimization of virtual mass placements, and it can find the global optimal solution;
3. A method of sensitivity analysis and error analysis is proposed and discussed for the purpose of determination of virtual mass value. The method guarantees that natural frequencies used for damage identification have high sensitivities to damage and a small identification error;
4. The problem of optimal sensor placement is similar to the optimal arrangement of virtual masses, so the basic theory of the proposed methods in this paper, including the arrangement of sensitivity matrix, Volumetric Maximum Criterion and improved PSO algorithm, can be studied further for optimal sensor placement of modal identification.

## Figures and Tables

**Table 1:** PSO Algorithm Parameter Settings.

Population Size	Particle Dimension	The Maximum Number of Iterations	Inertia Factor		Learning Factor		Random Number		Particle Velocity	
			*w*_max_	*w*_min_	*c*_1_	*c*_2_	*r*_1_	*r*_2_	*v*_max_	*v*_min_
40	19	100	0.9	0.4	1.496	1.496	[0–1]	[0–1]	4	4

**Table 2:** Random optimization results of discrete PSO.

Random Test Number	The Optimal Value	Optimal Fitness Value	Optimization Time
1~4 times	1, 3, 5, 8, 13, 15, 17, 19	−0.022	0.697 s~1 s

**Table 3:** First identified three natural frequencies when six virtual masses are arranged on the damaged structure/Hz.

DOF	1	3	8	12	17	19
First natural frequency	3.900	3.899	3.900	3.899	3.899	3.899
Second natural frequency	23.899	24.099	23.900	24.300	24.399	24.099
Third natural frequency	91.298	90.498	91.098	91.098	91.398	90.798

**Table 4:** First three theoretical frequencies when six virtual masses are added to the undamaged finite element model/Hz.

DOF	1	3	8	12	17	19
First natural frequency	3.899	4.199	4.000	3.999	3.900	3.899
Second natural frequency	23.900	23.999	23.899	23.899	24.399	24.399
Third natural frequency	92.398	92.398	92.398	92.398	91.898	92.498

**Table 5:** The first four natural frequencies of the undamaged and the damaged structure/Hz.

Frequency Order	1	2	3	4
Undamaged structure	37.7299	65.6261	120.2154	192.8448
Damaged structure	34.9979	63.8086	119.3401	188.4917

**Table 6:** Number and optimized locations of additional virtual masses.

Case	Number	Locations
1	4	7,8,9,13
2	5	7,9,10,13,15
3	6	7,8,9,13,14,15
4	7	7,9,10,11,13,14,15
5	8	7,8,9,10,11,13,14,15
6	9	1,7,8,9,10,11,13,14,15
7	10	1,5,7,8,9,10,11,13,14,15
